# Chlorophyll Fluorescence Imaging-Based Duckweed Phenotyping to Assess Acute Phytotoxic Effects

**DOI:** 10.3390/plants10122763

**Published:** 2021-12-14

**Authors:** Viktor Oláh, Anna Hepp, Muhammad Irfan, Ilona Mészáros

**Affiliations:** Department of Botany, Faculty of Science and Technology, University of Debrecen, Egyetem tér 1, H-4032 Debrecen, Hungary; pitypang666@gmail.com (A.H.); muhadirfan6167@gmail.com (M.I.); immeszaros@unideb.hu (I.M.)

**Keywords:** chlorophyll fluorescence imaging, PAM fluorometry, duckweed test, *Spirodela polyrhiza*, phytotoxicity, hormesis

## Abstract

Duckweeds (Lemnaceae species) are extensively used models in ecotoxicology, and chlorophyll fluorescence imaging offers a sensitive and high throughput platform for phytotoxicity assays with these tiny plants. However, the vast number of potentially applicable chlorophyll fluorescence-based test endpoints makes comparison and generalization of results hard among different studies. The present study aimed to jointly measure and compare the sensitivity of various chlorophyll fluorescence parameters in *Spirodela polyrhiza* (giant duckweed) plants exposed to nickel, chromate (hexavalent chromium) and sodium chloride for 72 h, respectively. The photochemistry of Photosystem II in both dark- and light-adapted states of plants was assessed via *in vivo* chlorophyll fluorescence imaging method. Our results indicated that the studied parameters responded with very divergent sensitivity, highlighting the importance of parallelly assessing several chlorophyll fluorescence parameters. Generally, the light-adapted parameters were more sensitive than the dark-adapted ones. Thus, the former ones might be the preferred endpoints in phytotoxicity assays. Fv/Fm, i.e., the most extensively reported parameter literature-wise, proved to be the least sensitive endpoint; therefore, future studies might also consider reporting Fv/Fo, as its more responsive analogue. The tested toxicants induced different trends in the basic chlorophyll fluorescence parameters and, at least partly, in relative proportions of different quenching processes, suggesting that a basic distinction of water pollutants with different modes of action might be achievable by this method. We found definite hormetic patterns in responses to several endpoints. Hormesis occurred in the concentration ranges where the applied toxicants resulted in strong growth inhibition in longer-term exposures of the same duckweed clone in previous studies. These findings indicate that changes in the photochemical efficiency of plants do not necessarily go hand in hand with growth responses, and care should be taken when one exclusively interprets chlorophyll fluorescence-based endpoints as general proxies for phytotoxic effects.

## 1. Introduction

Duckweed species (members of the Lemnaceae family) are widely used test objects in ecotoxicology research. In fact, phytotoxic effects of potential toxicants on aquatic macrophytes are presently modelled predominantly by using duckweeds (*Lemna minor* L., *L. gibba* L. and *Spirodela polyrhiza* L. Scleid.) in standardized ecotoxicity tests (e.g., OECD Guideline 221, ISO No. 20079 and ISO/NP No. 20227, respectively). They owe this popularity due to their small size, fast and predominantly vegetative reproduction, simple anatomy and sensitivity to various aquatic toxicants [1,2]. Duckweed ramets—usually referred to as fronds—basically consist of an upper and lower epidermis, a spongy mesophyll and one or two meristematic regions, which differentiate new fronds. The size of the fronds is in the mm–cm range and the doubling time of their cultures can be as fast as two days under suitable growth conditions [3]. In duckweed-based phytotoxicity assays, toxic effects have been usually quantified via the inhibition of biomass growth in cultures, in terms of frond number, frond area, fresh or dry mass change, respectively [4,5]. In the course of the methodological development, other test endpoints, such as chlorophyll- or N-content [6], colony disintegration [7], or root growth [8] have also been applied, but due to their simplicity and straightforward interpretation, the biomass-based toxicity endpoints became the most common parameters in *Lemna*-tests. In order to properly measure biomass growth inhibition, it is inevitable to ensure the test cultures grow for several frond generations. The most common ISO [4] and OECD [5] test protocols use seven-day-long exposures and require a control biomass doubling time shorter than 2.5 days in order to ensure adequate sensitivity of tests.

As a promising alternative to the biomass-based methods in duckweed phytotoxicity-tests, the applicability of *in vivo* chlorophyll fluorescence (ChlF) has been long studied [9]. ChlF predominantly originates from Photosystem II (PSII) as the fraction of absorbed light energy that has not been utilized in photochemical energy conversion nor released as heat. Photosynthesis provides the basis for plant growth and is a complex process with several sensitive components. Thus, ChlF supposedly can be used as an early-warning proxy of adverse effects, offering fast and sensitive phytotoxicity test protocols [10,11]. Pulse-amplitude modulated (PAM) fluorescence imaging is an emerging tool to assess phytotoxic effects [12]. It is based on acquiring fluorescence signals from 2 or three-dimensional objects via a CCD camera, and thus enables analyses on spatial heterogeneities in photosynthetic properties. This technique also allows parallel phenotyping of several samples under identical conditions, e.g., in multiwell plates.

Imaging PAM fluorometry has already been proven to be applicable in assessing phytotoxic effects in algae [13,14], seagrass [15,16], or mosses [17]. Duckweeds are apparently ideal objects for ChlF imaging-based phytotoxicity assays: they are easy to grow in genetically homogenous, aseptic cultures and are small enough to fit into multiwell plates. In addition, the flat fronds of most species float on the water surface facing towards the CCD camera while taking up toxicants directly to the assimilating tissue. The internationally standardized protocols and the already published, extensive duckweed phytotoxicity literature provides solid grounds for further methodological development. An increasing body of reports indicates that ChlF imaging-based duckweed phenotyping has been successfully applied in assessing the effects of various stressors (Table S1). In the studies listed in Table S1, ChlF was predominantly assessed by means of two commercially available platforms: the Imaging-PAM (Walz GmbH, Effeltrich, Germany, 22 out of 29 papers) and FluorCam (Photon System Instruments, Brno, Czech Republic, 6 out of 29 papers) instruments. The ChlF parameters were used either solely or in combination with other test endpoints derived from, e.g., plant growth, photosynthetic pigment content, or oxidative stress markers, and proved to be a robust proxy for phytotoxic effects in several cases. 

**Figure 1:**
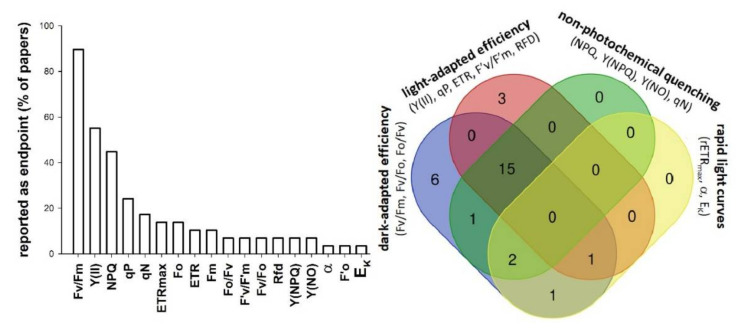
Reported application of various chlorophyll fluorescence induction-derived phytotoxicity test endpoints in scientific studies assessing duckweeds by means of chlorophyll fluorescence imaging (**left**), and Venn diagram denoting the number of studies using different types of chlorophyll fluorescence parameters as test endpoints (**right**). Details of the reviewed 29 papers are summarized in Supplementary Table S1.

PAM chlorophyll fluorescence measurements generate an excessive number of various parameters that are basically derived from five mutually independent chlorophyll fluorescence levels, that is ground (Fo) and maximal fluorescence yields (Fm) in the dark-adapted state; and steady-state (Fs), ground (F’o) and maximal fluorescence yields (F’m) in light-acclimated state of samples, respectively. The referred 29 papers in Figure 1 (for details, see Table S1) report a total of 18 ChlF-derived test endpoints -with a median of 4 parameters per study- of which Fv/Fm (90% of papers), Y(II) (55% of papers) and NPQ (45% of papers) were the most common ones. This diversity of potentially applicable test endpoints makes it difficult to compare ChlF-based phytotoxicity data from different sources. Additionally, most papers report data for single toxicants or well-defined groups of toxicants with similar modes of action (e.g., PSII inhibiting herbicides), but apply various exposure conditions. Thus, it is hard to generalize plant responses in terms of ChlF parameters, or to assess the specificity of particular ChlF endpoints to different toxicants. It can also be noted that, though most of the reviewed studies jointly used various types of chlorophyll fluorescence parameters, a significant part (~30%) of them relied exclusively on either dark- or light-adapted photochemical efficiency indices (Figure 1).

In the present study, we assessed the applicability of a ChlF imaging-based duckweed test method performed in multiwell tissue culturing plates as an alternative to the standard duckweed growth inhibition tests. In order to do this, we used three different toxicants, namely nickel (Ni), hexavalent chromium -Cr(VI)- and sodium chloride (NaCl), and parallelly analyzed concentration-response curves of several ChlF parameters. The applied toxicants have different modes of action, and they have also been suggested as reference toxicants in standard duckweed growth inhibition tests [1,5]. Ni is essential for plants as a cofactor for enzymes involved in N-metabolism. Ni thus promotes plant growth at low concentrations, but in high doses, it induces disorders in nutrient uptake, N-metabolism, and water balance, leading to growth inhibition and declining fitness in the longer term [18]. Ni at higher concentrations affects photosynthesis by decreasing chlorophyll content, altering chloroplast ultrastructure, displacing Mg in chlorophyll and RuBisCo, inhibiting the photosynthetic electron transport chain, and inactivating PSII and Photosystem I (PSI), respectively [19,20,21]. In aqueous environments, its prevalent oxidation form is Ni^2+^ ion, and it disturbs duckweed growth starting from the 10^−4^ g L^−1^ concentration range [22,23]. Chromium has no proven beneficial physiological role in plants [24]. Hexavalent form of chromium occurs in waters as CrO_4_^2−^ and Cr_2_O_7_^2−^ oxyanions, and those forms can be actively taken up by plants via the sulfate transport system [25]. Cr(VI) toxicity can lead to impaired nutrient balance, e.g., because of the inhibition of plasma membrane H^+^-ATPase, nitrate reductase, and Fe(III)-reductase enzymes. Cr(VI) can react with macromolecules in cells, and thus triggers oxidative stress while it gets reduced to Cr(III) [18]. At the photosynthesis level, Cr(VI) decreases chlorophyll content, inhibits the photosynthetic electron transport, inactivates enzymes of the Calvin–Benson-cycle, and disorganizes chloroplast ultrastructure, respectively [25]. According to Naumann et al. [22] and Oláh et al. [23], Cr(VI) inhibits duckweed growth in the 10^−3^–10^−4^ g L^−1^ range. NaCl, besides inducing osmotic stress, impairs plant growth by Na^+^ and Cl^-^ ion toxicity. Salinity stress disturbs mineral nutrition and water relations, alters protein conformation and triggers the formation of reactive oxygen species (ROS) [26]. NaCl was reported to reduce chlorophyll content, inhibit electron transport and change chloroplast ultrastructure [27]. It inhibits the growth of various duckweed species in the 10^0^–10^1^ g L^−1^ concentration range [28].

By applying the above toxicants, our specific aims were (i) to assess the concentration-response relationships of different ChlF-based test endpoints, (ii) to find the most responsive ones, and (iii) to test if different ChlF-parameters show general or rather toxicant-specific patterns in responses.

## 2. Materials and Methods

### 2.1. Plant Material and Treatments

The tests were performed with the giant duckweed (*Spirodela polyrhiza* (L.) Schleid.) clone UD0401 (RDSC #5501) [29]. Test plants were obtained from seven-day-old stock cultures maintained on 100 mL of Steinberg medium ([1] pH 6.0 ± 0.2) in 300 mL Erlenmeyer flasks under constant temperature (24 ± 2 °C) and irradiation (white, 58 ± 4 µE m^−2^ s^−1^).

The reference toxicants were applied in the following final concentrations:

Ni (as NiSO_4_ × 7H_2_O) or Cr(VI) (as K_2_Cr_2_O_7_): 0; 0.039; 0.078; 0.156; 0.313; 0.625; 1.25; 2.5; 5.0 and 10.0 mg L^−1^, respectively.

NaCl: 0; 2; 4; 6; 8; 10; 12; 14 and 16 g L^−1^, respectively.

The exposures were performed in 12-well tissue culture plates, and each well contained 4 mL test solution, i.e., Steinberg medium containing either toxicant at an above-listed concentration. At the beginning of tests, 1-1 healthy *S. polyrhiza* colony with 4–5 fronds were inoculated in each well. The exposures lasted for three days (72 ± 4 h). This duration was based on preliminary experiments, which indicated that ChlF parameters were not affected by nutrient depletion or crowding while frond numbers had doubled under control conditions.

### 2.2. Chlorophyll Fluorescence Imaging

After three days of toxicant treatments, the ChlF parameters of test plants were measured by means of Maxi Imaging PAM (Heinz Walz GmbH, Effeltrich, Germany) equipped with blue Imag-Max/L LED-array Illumination Unit (wavelength λ = 450 nm) and IMAG-K6 CCD camera (2/3″ chip with 1392 × 1040 pixels resolution) using the following protocol (for example fluorescence charts see also Supplementary Figure S1):

1. First, the test plants in plate were dark-adapted for 20 min in order to reach a fully oxidized state of PSII photochemistry (all PSII reaction centers were “open”).
2. In dark-adapted state ground fluorescence yield (Fo) of plants was determined using weak, non-inductive measuring light (blue, 450 nm, intensity: 2, frequency: 1).
3. A single saturating pulse (blue, 450 nm, intensity: 9, i.e., ~4000 µE m^−2^ s^−1^ with the applied instrumental setup, length 720 ms) was used to saturate PSII photochemistry (all PSII reaction centers became “closed”) and to determine maximal fluorescence yield (Fm) of plants.
4. After the saturation pulse, a continuous actinic irradiation (blue, 450 nm) with similar intensity (intensity: 5, equivalent to ~77 µE m^−2^ s^−1^ in the applied instrumental setup) to that of the plants’ ambient light environment was switched on for 10 min to induce a light-adapted state of plants (Supplementary Figure S1). After 10 min, steady-state chlorophyll fluorescence yield (Fs) of plants under actinic illumination was determined and a second saturating pulse—with the same settings as for determining Fm—was applied to measure their light-adapted maximal fluorescence yield (F’m). Due to technical reasons, F’o, which is the ground fluorescence yield in a light-acclimated state, was not directly measured by the instrument, but calculated using the formula [30]:

F’o = Fo/(Fv/Fm + Fo/F’m).

Using the above five, mutually independent ChlF yields, ChlF parameters were calculated according to Table 1.

Immediately after the termination of the above measuring sequence, a so-called rapid light curve (RLC) of PSII photochemistry was performed with 15 consecutively increasing irradiation steps (PPFD: 0-530 E m^−2^ s^−1^, each light step lasted for 30 s), using the built-in light curve protocol of the instrument. At each step, the electron transport rate (ETR) of plants was calculated according to the formula [30]:

ETR (μmol e^−^ m^−2^ s^−1^) = (ΔF/F’m) × PPFD × 0.5 × 0.84 (1)

where “∆F/F’m” was the effective quantum yield of photochemical energy conversion in PSII [i.e., Y(II)] at a given light intensity; “PPFD” is the nominal incident photon flux density in terms of µE m^−2^ s^−1^; “0.5” referred to an assumed stoichiometric distribution of absorbed light energy between the two Photosystems, and “0.84” denoted an empirical 84% absorption efficiency of incident light in higher plants [12,15,34,35].

By plotting the obtained ETR values as a function of the corresponding actinic irradiation intensities, a saturation curve was obtained that allowed the calculation of maximal photon use efficiency (α) and maximal ETR (ETR_max_) under the applied ambient conditions as follows: 

α was calculated as the initial slope of the curve at the first light step, i.e.:

α = ETR/11 µE m^−2^ s^−1^ (2)

ETR_max_ was considered as the highest calculated ETR value of the curve.

### 2.3. Data Processing and Statistical Analyses

Each concentration was applied in four parallel treatments (wells) and the experiments were repeated three times with the same experimental design. During data processing, means of the four parallels within an experiment were normalized to their respective control means, resulting in a total of n = 3 data per toxicant concentration. Concentration-dependent responses of Fv/Fm, Fv/Fo, Y(II), qP, F’v/F’m, F’v/F’o, Rfd, α and ETR_max_ were analyzed by fitting non-linear regression models to the data. Based on the assumption that all the above endpoints reflect the efficiency of PSII photochemistry in their specific way, and, theoretically, can decrease to zero, the concentration-dependence of ChlF parameters were assessed by means of log-logistic models fixing the lower limit of the functions to 0. Model fittings were performed by means of the “drc”-package (version 2.5-12) [36], in R statistical environment (version 3.2) [37], using “LL.3” (three-parameter log-logistic) function of the package. 

Additionally, plant responses were also modelled by the modified four-parameter log-logistic functions “BC.4” and “CRS.4” of the “drc”-package, respectively. These models were developed by [38] (“BC”-model) and by [39] (“CRS”-model) in order to include a hormetic component at the lower concentration range. After visually checking the model fittings, the goodness of fit for different regression models was compared based on the Akaike information criterion (AIC), using the “mselect” function of the “drc”-package. In those cases, where the hormetic component did not contain additional information based on the AIC score, the three-parameter log-logistic model was applied to the given endpoint. In those cases, on the other hand, when the hormetic models described the concentration-response relationships better, the model selection was based on the estimated residual standard error of the models by means of the “mselect” function. The selected model for a given endpoint was also checked by the lack-of-fit test using the “modelFit” function and tested by means of pseudo-R^2^ using the “cor” function of the “drc”-package, respectively. In the latter procedure, correlation between the predicted and observed responses in terms of each ChlF parameter was compared at the applied toxicant concentrations.

Based on the fitted regression models, the effective concentrations resulting in 20 and 50% inhibition of the given endpoint (EC_20_ and EC_50_, respectively) were estimated by means of the “ED” function of the “drc”-package. When hormetic models were used, the “No Observable Adverse Effect Concentration” (NOAEC), i.e., the toxicant concentration at which the hormetic effect diminishes; the maximum of the hormetic response (“max”), and the corresponding concentration at which the maximal hormetic response occurs (EC_max_) were also estimated using the “MAX” function of the “drc”-package.

## 3. Results

**Figure 2:**
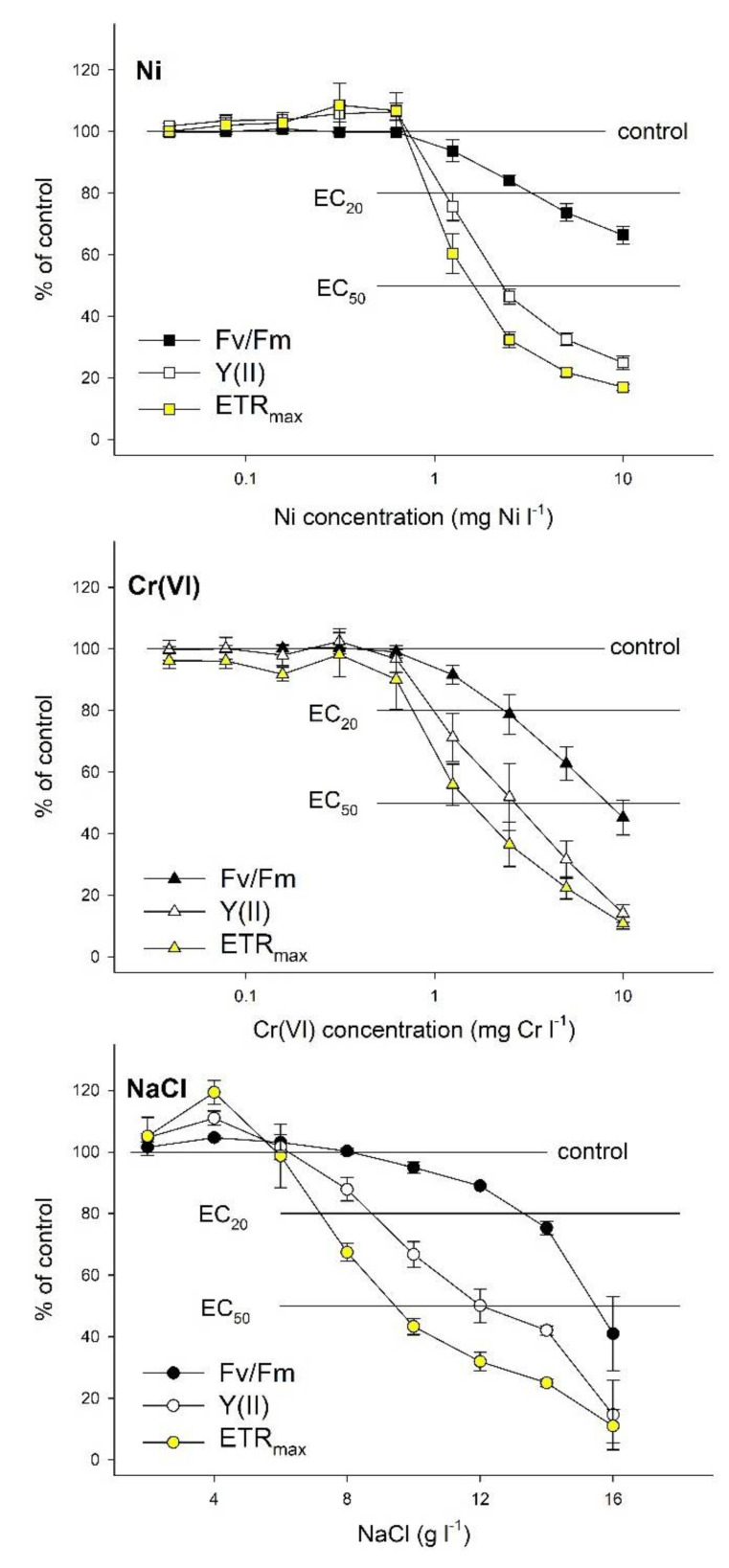
Concentration-response relationships of the dark-adapted (Fv/Fm) and light-adapted photochemical energy conversion -Y(II)- and of the maximal electron transport rate (ETR_max_) for *S. polyrhiza* UD0401 test plants exposed to Ni, Cr(VI) or NaCl for 72 h, respectively. Symbols denote grand means (means of means) ± SE of three experiments (n = 3), as a percentage of respective control data. For summary statistics of every measured parameter, please refer to Supplementary Tables S3, S5 and S7, respectively.

After 72 h-long exposures, all three toxicants affected the assessed ChlF parameters in the applied concentration range (Figure 2). In general, Ni has an inhibiting effect (>5%) from 1.25 mg L^−1^. From that concentration, the assessed indices of PSII efficiency decreased in a concentration-dependent manner (Figure 2, the summary statistics of the original data are supplemented in Table S2). On the other hand, below 1.25 mg L^−1^ Ni proved to have hormetic effect in the case of most parameters (Table 2, for the plotted model fittings, refer to Supplementary Figure S2). Though the maximal response did not exceed 105% of control in some cases, concentration-response relationships were better described by those non-linear models that included the hormetic component, with the only exception of that of Fv/Fm. The maximal hormetic response exceeded the control by 10% in the case of maximal electron transport rate (ETR_max_). In general, the NOAEC ranged between 0.6–0.7 mg L^−1^ and the Ni concentration resulting in maximal hormetic response was 20–40% of that concentration (0.1–0.3 mg L^−1^, Table 2). By comparing the obtained EC_20_ and EC_50_ values, we found considerable differences amongst different parameters. The calculated EC_20_ values were in the 1.0–3.7 mg L^−1^ range while EC_50_ values ranged from 1.8 to 20.6 mg L^−1^, and four of the assessed nine parameters had higher calculated EC_50_ than the maximal applied Ni-concentration (Table 2). ETR_max_ and the effective quantum yield of photochemical energy conversion in a light-adapted state [Y(II)] responded with the highest sensitivity (i.e., lowest EC_20_ and EC_50_) while the maximum quantum yield of PSII photochemistry (Fv/Fm) and the relative fluorescence decrease (Rfd) proved to be the least responsive parameters, respectively.

Cr(VI)-induced inhibition started from 1.25 mg L^−1^ for most ChlF parameters, though ETR_max_ and the ratio of quantum yields of photochemical and concurrent non-photochemical processes in PSII in the light-adapted state (F’v/F’o) showed ~10% decrease even at 0.625 mg L^−1^ (Figure 2, the summary statistics of the original data are supplemented in Table S3). In contrast with Ni, Cr(VI) did not result in hormetic response (Table 3). Though concentration-response relationships for most ChlF parameters were better described by models including a hormetic component than by the three-parameters log-logistic model, the calculated maxima did not exceed 102% of the control with the only exception of Rfd (Table 3, for the plotted model fittings, refer to (Supplementary Figure S3). The calculated EC_20_ ranged from 0.7 to 2.5 mg L^−1^ while EC_50_s were in the 1.8–9.0 mg L^−1^ range. Similar to Ni, the lowest EC_20_ and EC_50_ was calculated for ETR_max_, while the highest effective concentrations were found in cases of Rfd and Fv/Fm (Table 3).

Different ChlF parameters showed diverging responses to NaCl-treatments too. Photochemical quenching (qP) and Rfd started to decrease from 6 g L^−1^ NaCl, Y(II) and ETR_max_ from 8 g L^−1^, while Fv/Fm, Fv/Fo, F’v/F’m (i.e., the effective quantum yield of PSII photochemistry in the light-adapted state), F’v/F’o and the maximal photon use efficiency (i.e., α) decreased from 10 g L^−1^ NaCl, after 72 h of exposure (Figure 2, the summary statistics of the original data are supplemented in Table S4). Several parameters showed distinct hormetic responses to lower NaCl concentrations, including Fv/Fo, Y(II), F’v/F’o, and ETR_max_, respectively (Figure 2 and Figure S4, Table 4). The maximal stimulation ranged from 108 to 125% of respective control data with calculated EC_max_ of 3.2–4.6 g L^−1^ that is 50–57% of the calculated NOAEC. It should also be noted that even those parameters which were better described by three-parameter log-logistic functions increased by 2−7% at lower (2−4 g L^−1^) concentrations (data not shown). The calculated EC_20_ concentrations were in the 7.4−13.2 g L^−1^ range, while EC_50_ was between 9.7 and 15.5 g L^−1^ for the assessed endpoints (Table 4). Similar to the heavy metal treatments, ETR_max_ showed the highest sensitivity, and Fv/Fm and Rfd were the least responsive parameters.

**Figure 3:**
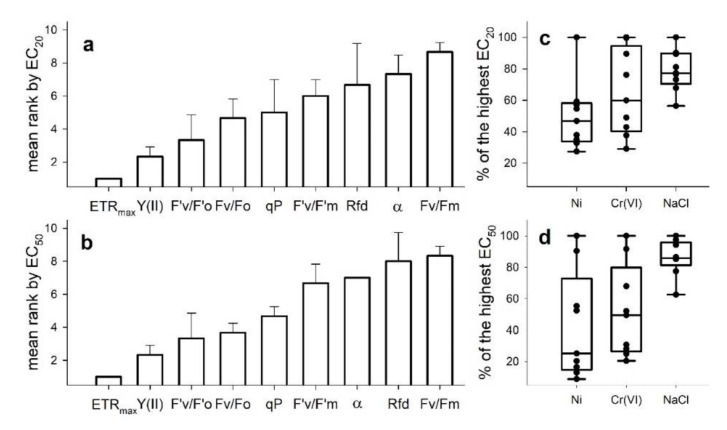
Responsivity of the assessed ChlF-based test endpoints as their mean relative sensitivity ranks (± SE, n = 3, subfigures (**a**) for EC_20_ and (**b**) for EC_50_). Relative ranks were based on the calculated effective Ni, Cr(VI) and NaCl concentrations resulting in 20 (EC_20_) and 50% (EC_50_) inhibition of the respective parameter after 72 h long exposures. Variability in the calculated EC_20_ and EC_50_ concentrations for the assessed ChlF parameters (subfigures (**c**) for EC_20_, (**d**) for EC_50_) were normalized to the highest respective calculated effective concentration in the case of each applied toxicant.

In order to compare the overall sensitivity of the assessed tests endpoints, we used mean ranks based on their calculated EC_20_ and EC_50_ values for the three toxicants, respectively. The ranks indicated a consistent order of sensitivity with the lowest effective concentrations calculated for ETR_max_, Y(II) and F’v/F’o while Fv/Fm proved to be the least sensitive parameter (Figure 3a,b). Even Fv/Fo, which is based on the same two basic ChlF yields, i.e., ground (Fo) and maximal (Fm) fluorescence yields, resulted in lower effective concentrations as compared to Fv/Fm, or the light acclimated test endpoints qP, F’v/F’m, Rfd and α. The concentration ranges for the obtained EC_20_ and EC_50_ values were considerably wider in the case of the two tested heavy metals, while effective concentrations scattered in a much narrower range when NaCl was analyzed (Figure 3c,d).

**Figure 4:**
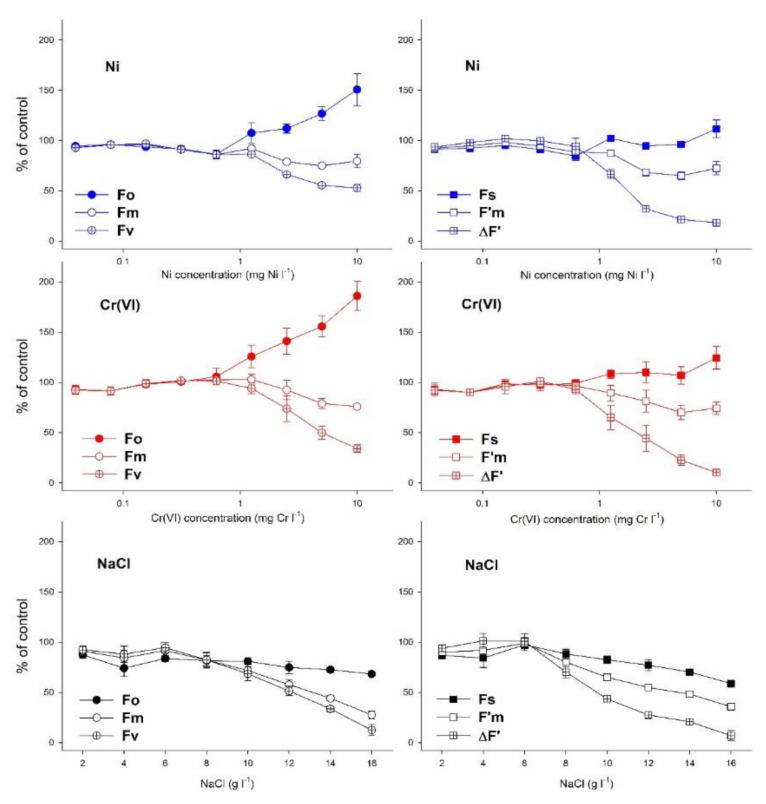
Changes in the dark-adapted (Fo, Fm, **left**) and light-adapted (Fs, F’m, **right**) basic ChlF yields and the derived variable fluorescence parameters (Fv, ∆F’) after 72 h long exposures to Ni, Cr(VI) and NaCl, respectively. Symbols denote grand means (means of means) ± SE of three experiments (n = 3), as a percentage of respective control data. For summary statistics of every measured parameter, please refer to Supplementary Table S5.

Concentration-response relationships of the basic ChlF parameters determining the variable fluorescence Fv in dark-adapted (Fo, Fm) and ∆F in the light-adapted state (Fs, F’m) indicated different patterns for heavy metals as compared to NaCl (Figure 4, the summary statistics of the original data are supplemented in Table S5). Ni and Cr(VI) reduced Fv and ∆F via a parallel increase in Fo and Fs and a decrease in Fm and F’m, respectively. It should also be noted that Fo showed a considerably larger percentile increase due to heavy metal treatments (151 and 186% of control at 10 mg L^−1^ Ni and Cr(VI) compared to Fs (111 and 124% of control at 10 mg L^−1^ Ni and Cr(VI), Figure 4). Fm and F’m, on the other hand, were similarly affected: 10 mg L^−1^ Ni and Cr(VI) decreased Fm by 20 and 24% compared to control, while F’m was lowered by 28 and 25%, respectively (Figure 4). Contrary to the tested heavy metals, NaCl decreased all ChlF yields (Fo, Fm, Fs and F’m) in a concentration-dependent manner, and inhibited the calculated quantum efficiencies through reducing maximal fluorescence parameters (i.e., Fm and F’m) stronger as compared to Fo and Fs (Figure 4).

**Figure 5:**
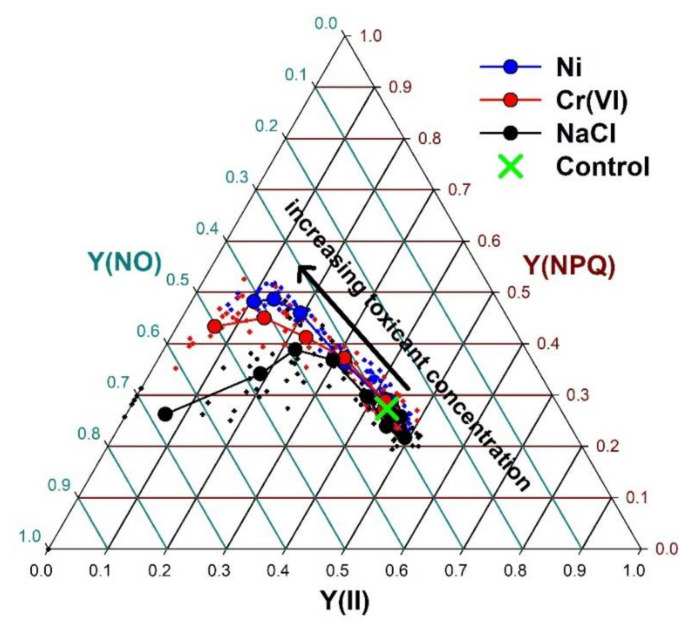
Changes in the relative ratios of photochemical -Y(II), regulated non-photochemical -Y(NPQ), and non-regulated non-photochemical -Y(NO)- quenching along the increasing Ni, Cr(VI) and NaCl concentrations, respectively. Small dots denote original data, large symbols with corresponding colors denote means of 12 samples at a given toxicant concentration.

When proportions of chlorophyll fluorescence quenching processes were compared, similar response patterns were found for different toxicants in the lower concentration ranges (Figure 5). Compared to the control, low concentrations did not change the ratio of the effective quantum yield of PSII photochemical energy conversion [Y(II)], the quantum yield of regulated PSII non-photochemical energy loss (Y(NPQ)) and the quantum yield of non-regulated heat dissipation and fluorescence emission (Y(NO)); or even resulted in a slight increase in Y(II), as a hormetic response, with a parallel decrease in Y(NPQ). Increasing concentrations of the two heavy metals, however, resulted in a gradual decline in Y(II), in parallel with an increase in Y(NPQ), and also slightly elevated Y(NO) at the highest concentrations (Figure 5). Contrary to them, NaCl-induced increase in Y(NPQ) was only intermittent (up to 12 g L^−1^) and was followed by a decline in that parameter with parallel increase in Y(NO) (Figure 5).

## 4. Discussion

Our results indicate that acute phytotoxicity of various chemicals can be efficiently characterized via the ChlF imaging-based duckweed phenotyping method. One of the crucial points is the proper choice of the most responsive ChlF parameters as test endpoints [35]. The sensitivity of the assessed ChlF parameters was found to be highly variable with respect to a given toxicant used in our study. The difference between the endpoints with the lowest and highest calculated EC_50_-s was 11-fold, 5-fold and 1.6-fold in the case of Ni, Cr(VI) and NaCl, respectively. In general, Fv/Fm and Rfd proved to be the least sensitive endpoints. Rfd has been applied widely in plant ecophysiology studies, while Fv/Fm is by far the most basically reported ChlF parameter literature-wise. Our results indicate that one should be cautious with relying exclusively on the latter parameter in toxicological studies. Fv/Fo resulted in considerably lower effective concentrations, though the calculation of this parameter uses the same basic ChlF levels, i.e., Fo and Fm, as Fv/Fm. Due to the different mathematical basis, Fv/Fo has higher values and performs a larger dynamic range than Fv/Fm [33]. Consequently, the relationship between these two endpoints is non-linear and results in higher sensitivity of Fv/Fo in the physiologically near-optimum range [31,40,41,42,43].

Differences in the photochemical efficiency of plants can be better distinguished if actinic irradiation is applied, as the limiting steps of photochemistry are put under pressure in this way [10,44,45,46,47,48]. Our results confirmed those previous observations: Y(II) was a very responsive endpoint, and both F’v/F’m and F’v/F’o exhibited lower calculated effective concentrations as compared to Fv/Fm and Fv/Fo, respectively. ETR_max_ had also been reported as a very sensitive endpoint in detecting herbicide and heavy metal toxicity in many previous duckweed tests [34,42,44,49,50,51]. From this aspect, the more pressure seems to be the better, as maximum electron transport rate (ETR_max_) proved to be the most sensitive parameter to each toxicant in our study. ChlF imaging-based assays generate detailed datasets both in terms of spatial resolution and different aspects of photosynthetic processes [11]. Besides the above indices for photochemical efficiency, basic ChlF parameters (Fo, Fm, F’o and F’m) or indicators of non-photochemical quenching (Y(NPQ), Y(NO), NPQ, and qN) can also be useful to characterize particular effects of various toxicants [44,45,52]. Comparing the effects of the two heavy metals and NaCl, we found different patterns of changes in ChlF parameters along with increasing toxicant concentrations. These results underline the importance of analyzing additional ChlF parameters besides the most commonly reported Fv/Fm and Y(II). Changes in non-photochemical quenching can be seen as a sensitive indicator of physiological changes as Y(II), and may provide important additional data for understanding the phytotoxic effects of the applied toxicants [32,48,53].

**Figure 6:**
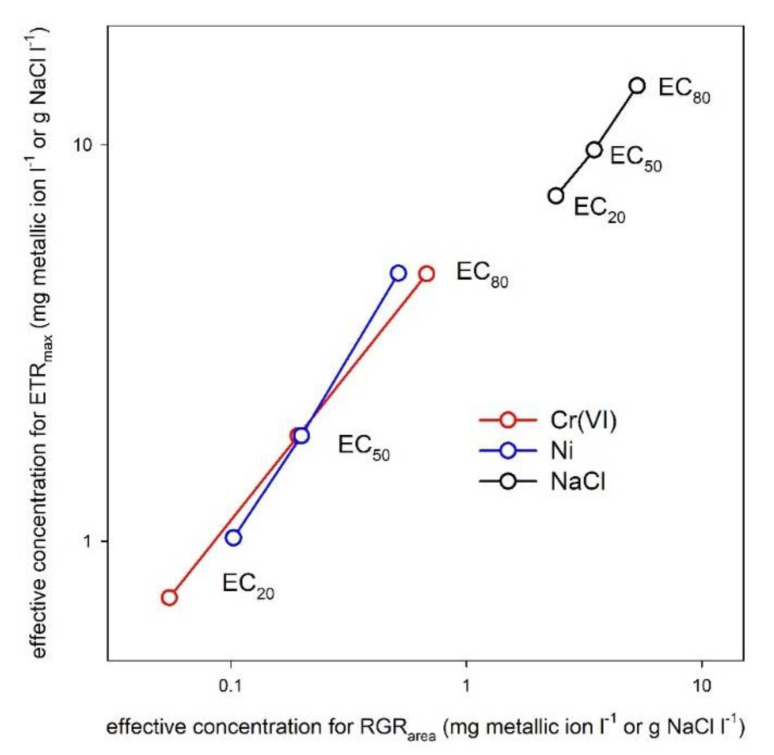
Correlations of the 20, 50 and 80% inhibiting concentrations (EC_20_, EC_50_ and EC_80_) of Cr(VI), Ni and NaCl were calculated for frond area-based relative growth rates (RGR_area_) in seven-day-long duckweed growth tests, and for maximal electron transport rates (ETR_max_) measured in three-day-long multiwell-based phytotoxicity tests with the *S. polyrhiza* clone UD0401, respectively. The effective concentrations for frond area growth inhibition were based on the data reported by Oláh et al. [23] and Hepp et al. [54], respectively.

ChlF parameters are considered to have comparable sensitivity to growth-based endpoints in aquatic plants [9,10]. In duckweed tests conducted with herbicide and phenol treatments for seven and three days, respectively, Fv/Fm and ETR_max_ showed lower calculated effective concentrations than the relative growth rates [44,52]. Our results did not support those former observations. Irrespective of their relatively high sensitivity, the calculated EC_50_s for ETR_max_ and Y(II) was considerably higher in the present study as compared to those calculated in growth inhibition tests using the same *S. polyrhiza* clone (clone UD0401, Figure 6). In the case of Ni and Cr(VI), the calculated EC_50_-s for frond area- and frond number-based growth rates were in the 0.18−0.2 mg L^−1^ range [23], which is one order of magnitude lower than the respective concentrations for ETR_max_. 

In NaCl treatments, the calculated EC_50_s were 3.45 and 4.51 g L^−1^ for frond area and frond number growth rates, respectively [54], which is one-half to one-third of that of ETR_max_ in the present study. It should be noted, however, that in the present study, relative growth rates were not determined, but obtained from a different experimental setup, that is, the OECD-conform [5] duckweed growth inhibition test. Thus, a possible reason for such diverging sensitivities can be the different treatment volume and duration: the growth inhibition tests were performed in 100 mL volume for seven days, while toxicant exposures in the present study were conducted in smaller medium volume (4 mL) and shorter exposure time (three days). This latter experimental setup thus may result in exposure to smaller doses on a biomass basis at the same nominal toxicant concentrations, and for a shorter duration as compared to the growth tests. Extended exposure times can drastically increase the sensitivity of phytotoxicity assays: in Cd-treated cultures of the same *S. polyrhiza* clone and under the same experimental conditions, three-day-long exposures resulted in three times higher calculated EC_50_-s of growth rate compared to the seven-day-long treatments [55]. 

Another explanation of the higher effective concentrations can be that growth rates reflect the overall performance of test plants under toxicant treatments. ChlF-based endpoints, on the other hand, reflect the operability of certain physiological processes, and if those are not affected directly, their response can be weaker and/or delayed [9,56]. Growth conditions might also be of crucial importance: test plants exposed to toxicants under sub-saturating irradiance—as was the case in the present study—have a better chance to up-regulate PSII repair systems and ROS scavenging than plants grown under higher irradiance levels [16,57]. This factor can further reduce the sensitivity of ChlF-based test endpoints compared to those derived from growth parameters.

Hormesis has been discussed extensively as a potentially general response to moderate environmental stress [58,59]. Hormetic-type responses were reported in duckweed toxicity tests conducted with organic and inorganic pollutants [39,60,61,62,63]. On the other hand, hormesis in photosynthetic responses of duckweeds is still scarcely studied [64]. Our results indicate that hormesis might be a frequent response of ChlF-based endpoints, at least in the shorter term. Based on the tested regression models, Ni and NaCl had a definite stimulating effect in case of several ChlF parameters in the low concentration ranges. The results suggest that, in general, the more sensitive parameters (Fv/Fo, Y(II), qP, F’v/F’o, ETR_max_) were more likely to indicate hormesis induced by Ni and NaCl treatments. For Cr(VI), hormetic models also described the concentration-response relationships better for several ChlF parameters. However, the maximal stimulation generally stayed below 3% in these cases, which is lower than the 5% limit, commonly defined as a criterion for hormetic responses [60]. The only exception was Rfd, which is the least sensitive endpoint in Cr(VI) treatments—which increased by a maximum of 5% as compared to control. 

The concentrations needed for the maximal hormetic response (EC_max_) were in the range of 2–15% of that of EC_50_ for Ni and 25–35% for NaCl, respectively. This observation was similar to that of [39], who found that stimulating concentrations usually fell in the 20–25% range of the EC_50_ concentrations in aquatic macrophytes treated with the herbicide terbuthylazine. The maximal response ranged in the 105–110% (Ni) and 110–125% (NaCl) of the respective control, similarly to earlier observations on plant hormetic responses [58,64]. Ni is essential for plants and Steinberg’s medium does not contain directly added Ni. Thus, Ni-induced stimulation of plant metabolism can be explained by its physiological role [18]. Similarly, NaCl was reported to enhance plant photosynthesis at low concentrations [26]. More interestingly, we found that stimulating Ni and NaCl concentrations for ChlF parameters in the present study were in the range of growth-inhibiting EC_50_ concentrations calculated in seven-day-long growth tests with the same *S. polyrhiza* clone [23,54]. These differences indicate that ChlF- and growth rate-based test endpoints do not implicitly describe the same phytotoxic pattern within the same concentration range, and one should be cautious when relying exclusively on ChlF-based endpoints as a general proxy for phytotoxic effects.

## 5. Conclusions

Chlorophyll fluorescence imaging-based duckweed phenotyping offers a non-destructive, fast, and easy way to assess the potential phytotoxicity of water pollutants, resulting in high throughput systems. Our results indicated that different chlorophyll fluorescence induction parameters responded with very different sensitivity to the applied treatments. In general, parameters measured in the dark-adapted state of test plants proved to be less sensitive than those measured in the light-adapted state. Thus, the latter ones might be the preferred endpoints in phytotoxicity assays. On the other hand, we also found that dark-adapted chlorophyll fluorescence parameters can result in different calculated effective concentrations, and Fv/Fo showed considerably higher responsivity than Fv/Fm. 

In future studies, it would be important to take this aspect into account and to report information on the Fv/Fo parameter as well. This might also be encouraged by the manufacturers by including this parameter in the default ones calculated by their instruments, as presently it’s missing from the repertoire of the most widely used Imaging-PAM platform. These results highlight the importance of parallel assessment of as many chlorophyll fluorescence parameters as possible, besides the most commonly reported Fv/Fm, Y(II), or NPQ. This finding was also supported by the observation that the tested toxicants induced different trends in the basic chlorophyll fluorescence parameters and, at least partly, in relative proportions of different quenching processes. Based on those differences, a basic distinction of water pollutants with different modes of action seems to be achievable by using this method. In the applied test protocol, the calculated effective concentrations proved to be higher than those calculated in growth inhibition tests performed with the same duckweed clone. Moreover, definite hormetic trends were found in responses of several endpoints in those concentration ranges where the applied toxicants resulted in strong growth inhibition in longer-term exposures. These differences suggest that changes in the photochemical efficiency of plants do not necessarily go hand in hand with growth responses, and care should be taken when one interprets exclusively ChlF-based endpoints as general proxies for phytotoxic effects.

## Figures and Tables

**Table 1:** The calculated ChlF parameters in dark-adapted and light-adapted states of *S. polyrhiza* test plants, measured after 20 min of dark-adaption and 10 min of non-saturating actinic irradiation, respectively.

Parameter	Calculation	Description	Reference
**Fv/Fm**	(Fm − Fo)/Fm	Maximum quantum yield of PSII photochemistry	[31]
**Fv/Fo**	(Fm − Fo)/Fo	Maximum ratio of quantum yields of photochemical and concurrent non-photochemical processes in PSII in dark-adapted state	[31]
**Y(II)**	∆F/F’m	The effective quantum yield of photochemical energy conversion in PSII	[31]
**Y(NPQ)**	(Fs/F’m) − (Fs/Fm)	Quantum yield of regulated non-photochemical energy loss in PSII	[32]
**Y(NO)**	Fs/Fm	Quantum yield of non-regulated heat dissipation and fluorescence emission	[32]
**qP**	1 − ((Fs − F’o)/F’v) = ∆F/F’v = (F’m − Fs)/(F’m − F’o)	Photochemical quenching of variable ChlF, i.e., the fraction of open PSII reaction centers in light-adapted state	[31]
**F’v/F’m**	(F’m − F’o)/F’m	Effective quantum yield of PSII photochemistry in light-adapted state	[31]
**F’v/F’o**	(F’m − F’o)/F’o	Effective ratio of quantum yields of photochemical and concurrent non-photochemical processes in PSII related to light-adapted state	[31]
**Rfd**	(Fm − Fs)/Fs	Chlorophyll fluorescence decrease ratio proportional to net photosynthesis; a.k.a. “vitality index”	[33]

**Table 2:** Results of non-linear regression model fittings to the assessed ChlF parameters after 72 h of Ni-treatments. Models “LL.3”, “BC.4” and “CRS.4” denote three-parameter log-logistic, 4-parameter Brain-Cousens and 4-parameter Cedergreen-Ritz-Streibig models, respectively. In the case of hormetic models, “max” denotes the highest calculated hormetic response as percent of the respective control data, “EC_max_” denote the Ni-concentration resulting in maximal hormetic response, and “NOAEC” denotes the “No Adversible Effect Concentration”, that is the Ni-concentration at which the hormetic effect diminishes. Bold numbers highlight maximal hormetic responses higher than 105%. EC_20_ and EC_50_ denote Ni-concentrations (as mg L^−1^ ± 95% confidence interval) resulting in 20 and 50% inhibition of the respective parameter. RSS and pseudo-R^2^ indicate the residual sum of squares and the correlation between the predicted and observed responses at the applied concentrations, respectively.

Parameter	Model	DF	RSS	F-Value	Pseudo-R^2^	Max	EC_max_	NOAEC (95% CI)	EC_20_ (95% CI)	EC_50_ (95% CI)
**Fv/Fm**	LL.3	27	323.56	1.7206	0.9286	NA	NA	NA	3.72 (2.87–4.57)	18.6 * (13.8–23.4)
**Fv/Fo**	BC.4	26	914.79	0.5439	0.9522	102.5	0.20	0.52 (0.16–0.88)	1.41 (1.10–1.72)	4.20 (3.36–5.03)
**Y(II)**	BC.4	26	679	3.6103	0.9775	**108.3**	**0.26**	**0.67** **(0.49–0.84)**	1.22 (1.05–1.38)	2.68 (2.35–3.01)
**qP**	CRS.4c	26	1652	1.0886	0.9151	**105.8**	**0.13**	**0.61** **(0.09–1.14)**	1.74 (1.14–2.35)	5.19 (3.81–6.56)
**F’v/F’m**	BC.4	26	404.32	1.4081	0.9600	102.6	0.21	0.61 (0.30–0.91)	2.03 (1.65–2.41)	10.8 * (7.55–14.0)
**F’v/F’o**	BC.4	26	1051.19	1.3835	0.9554	**105.6**	**0.23**	**0.64** **(0.38–0.89)**	1.3 (1.04–1.56)	3.40 (2.74–4.07)
**Rfd**	BC.4	26	302.72	3.8371	0.9602	103.4	0.13	0.38 (0.05–0.71)	2.13 (1.69–2.57)	20.59 * (12.0–29.2)
**α**	BC.4	26	747.14	0.3612	0.9267	103.3	0.23	0.68 (0.25–1.11)	2.20 (1.66–2.75)	11.4 * (6.70–16.1)
**ETR_max_**	BC.4	26	1671.24	2.5494	0.9600	**110.0**	**0.28**	**0.66** **(0.47–0.85)**	1.02 (0.85–1.19)	1.84 (1.56–2.13)

* Denote extrapolated effective concentrations, exceeding the highest applied Ni concentration (10 mg L^−1^). NA indicates that the respective parameter is not applicable due to a lack of hormetic response. The summary statistics of the original data and the fitted regression models are supplemented in Table S2 and Figure S2.

**Table 3:** Results of non-linear regression model fittings to the assessed ChlF parameters after 72 h of Cr(VI)-treatments. Models “LL.3”, “BC.4” and “CRS.4” denote three-parameter log-logistic, 4-parameter Brain-Cousens and 4-parameter Cedergreen-Ritz-Streibig models, respectively. In the case of hormetic models, “max” denote the highest calculated hormetic response as a percent of the respective control data, “EC_max_” denote the Cr(VI)-concentration resulting in maximal hormetic response, and “NOAEC” denote the “No Adversible Effect Concentration”, that is the Cr(VI)-concentration at which the hormetic effect diminishes. Bold numbers highlight maximal hormetic responses higher than 105%. EC_20_ and EC_50_ denote Cr(VI)-concentrations (as mg L^−1^ ± 95% confidence interval) resulting in 20 and 50% inhibition of the respective parameter. RSS and pseudo-R^2^ indicate the residual sum of squares and the correlation between the predicted and observed responses at the applied concentrations, respectively.

Parameter	Model	DF	RSS	F-Value	Pseudo-R^2^	Max	EC_max_	NOAEC (95% CI)	EC_20_ (95% CI)	EC_50_ (95% CI)
**Fv/Fm**	BC.4	26	727.5	0.0317	0.9336	100.5	0.18	0.45 (−0.58–1.47)	2.46 (1.89–3.03)	8.26 (6.25–10.3)
**Fv/Fo**	BC.4	26	1783.9	0.0571	0.9382	102.1	0.21	0.49 (−0.02–1.00)	1.21 (0.89–1.53)	2.77 (2.24–3.30)
**Y(II)**	CRS.4b	26	1873.8	0.2508	0.9395	102.2	0.24	0.4 (−0.03–0.83)	1.06 (0.72–1.40)	2.54 (2.00–3.09)
**qP**	LL.3	27	5191.1	0.3166	0.8252	NA	NA	NA	2.21 (1.13–3.23)	4.47 (3.26–5.68)
**F’v/F’m**	BC.4	26	984.35	0.066	0.9426	100.3	0.13	0.32 (−0.36–1.00)	1.48 (1.09–1.87)	4.70 (3.74–5.67)
**F’v/F’o**	BC.4	26	2127.9	0.1227	0.9307	100.9	0.14	0.33 (−0.16–0.82)	0.93 (0.63–1.23)	2.25 (1.75–2.74)
**Rfd**	BC.4	26	1537.2	0.2505	0.8784	**105.3**	**0.34**	**0.98** **(0.37–1.58)**	2.47 (1.77–3.17)	9.02 (5.12–12.9)
**α**	LL.3	27	918.75	0.2719	0.9357	NA	NA	NA	1.88 (1.34–2.43)	6.13 (5.11–7.15)
**ETR_max_**	LL.3	27	2303.1	1.0996	0.9344	NA	NA	NA	0.72 (0.47–0.98)	1.85 (1.45–2.24)

NA indicates that the respective parameter is not applicable due to a lack of hormetic response. The summary statistics of the original data and the fitted regression models are supplemented in Table S3 and Figure S3.

**Table 4:** Results of non-linear regression model fittings to the assessed ChlF parameters after 72 h of NaCl treatments. Models “LL.3”, “BC.4” and “CRS.4” denote three-parameter log-logistic, 4-parameter Brain-Cousens and 4-parameter Cedergreen-Ritz-Streibig models, respectively. In the case of hormetic models, “max” denotes the highest calculated hormetic response as a percent of the respective control data, “EC_max_” denote the NaCl-concentration resulting in maximal hormetic response, and “NOAEC” denotes the “No Adversible Effect Concentration”, that is the NaCl-concentration at which the hormetic effect diminishes. Bold numbers highlight maximal hormetic responses higher than 105%. EC_20_ and EC_50_ denote NaCl concentrations (as g L^−1^ ± 95% confidence interval), resulting in 20 and 50% inhibition of the respective parameter. RSS and pseudo-R^2^ indicate the residual sum of squares and the correlation between the predicted and observed responses at the applied concentrations, respectively.

Parameter	Model	Df	Rss	F-Value	Pseudo-R^2^	Max	EC_max_	NOAEC (95% CI)	EC_20_ (95% CI)	EC_50_ (95% CI)
**Fv/Fm**	LL.3	24	1109.59	0.5767	0.8996	NA	NA	NA	13.2 (12.4–14.0)	15.5 (15.0–15.9)
**Fv/Fo**	CRS.4a	23	1273.78	2.8564	0.9554	**113.2**	**4.55**	**8.78** **(7.47–10.1)**	10.7 (9.96–11.5)	13.2 (12.6–13.8)
**Y(II)**	CRS.4a	23	1771.4	1.3314	0.9381	**108.2**	**3.20**	**6.48** **(4.34–8.61)**	8.95 (7.87–10.0)	12.0 (11.1–12.8)
**qP**	LL.3	24	2319.2	1.4362	0.8816	NA	NA	NA	9.65 (8.13–11.2)	13.3 (12.3–14.3)
**F’v/F’m**	LL.3	24	1546.45	1.983	0.8844	NA	NA	NA	11.8 (10.7–13.0)	15.1 (14.4–15.9)
**F’v/F’o**	BC.4	23	2031	3.5422	0.9333	**124.6**	**4.63**	**8.57** **(7.52–9.61)**	10.2 (9.37–11.1)	13.3 (12.2–14.3)
**Rfd**	LL.3	24	1206.4	1.4771	0.9374	NA	NA	NA	10.1 (9.09–11.2)	13.4 (12.8–14.0)
**α**	LL.3	24	1479	0.8038	0.9053	NA	NA	NA	11.9 (10.9–12.9)	14.6 (14.1–15.2)
**ETR_max_**	BC.4	23	1632.4	0.9605	0.9595	**115.8**	**3.45**	**6.06** **(5.12–7.00)**	7.44 (6.76–8.11)	9.71 (9.06–10.4)

NA indicates that the respective parameter is not applicable due to a lack of hormetic response. The summary statistics of the original data and the fitted regression models are supplemented in Table S4 and Figure S4.

## Data Availability

The datasets used in the present study are available from the corresponding author on reasonable request.

## Supplementary Materials

The following is available online at https://www.mdpi.com/article/10.3390/plants10122763/s1, Table S1: Literature reports on the application of chlorophyll fluorescence imaging method in duckweed ecotoxicology and ecophysiology studies [65,66,67,68,69,70,71,72,73,74,75,76,77,78,79,80,81]. Table S2: Summary statistics of the assessed chlorophyll fluorescence induction parameters after 72 h-long Ni-treatments of the S. polyrhiza UD0401 clone. The table summarizes minimums (Min), maximums (Max), arithmetic means (Mean), standard deviations (SD) and coefficients of variation (CV) of pooled data, expressed as percentage of their respective control means, from 3 independent experiments with 4-4 parallel treatments at each applied Ni concentraiton (n = 12). Different upper cases indicate significantly (*p* < 0.05) different medians for different concentrations according to the Kruskal-Wallis test and post hoc Mann-Whitney pairwise comparisons. Table S3: Summary statistics of the assessed chlorophyll fluorescence induction parameters after 72 h-long Cr(VI)-treatments of the S. polyrhiza UD0401 clone. The table summarizes minimums (Min), maximums (Max), arithmetic means (Mean), standard deviations (SD) and coefficients of variation (CV) of pooled data, expressed as percentage of their respective control means, from 3 independent experiments with 4-4 parallel treatments at each applied Cr(VI) concentraiton (n = 12). Different upper cases indicate significantly (*p* < 0.05) different medians for different concentrations according to the Kruskal-Wallis test and post hoc Mann-Whitney pairwise comparisons. Table S4: Summary statistics of the assessed chlorophyll fluorescence induction parameters after 72 h-long NaCl-treatments of the S. polyrhiza UD0401 clone. The table summarizes minimums (Min), maximums (Max), arithmetic means (Mean), standard deviations (SD) and coefficients of variation (CV) of pooled data, expressed as percentage of their respective control means, from 3 independent experiments with 4-4 parallel treatments at each applied NaCl concentraiton (n = 12). Different upper indicate significantly (*p* < 0.05) different medians for different concentrations according to the Kruskal-Wallis test and post hoc Mann-Whitney pairwise comparisons. Table S5: Summary statistics of the assessed basic chlorophyll fluorescence induction parameters after 72 h-long treatments of the S. polyrhiza UD0401 clone to Ni, Cr(VI) and NaCl, respectively. The table summarizes minimums (Min), maximums (Max), arithmetic means (Mean), standard deviations (SD) and coefficients of variation (CV) of pooled data expressed as percentage of their respective control means, from 3 independent experiments with 4-4 parallel treatments at each applied concentration (n = 12). Different upper cases indicate significantly (*p* < 0.05) different medians for different concentrations according to the Kruskal-Wallis test and post hoc Mann-Whitney pairwise comparisons. Figure S1: Kinetics in the chlorophyll fluorescence yield of the S. polyrhiza UD0401 clone during the first saturation pulse after 20 min of dark adaption, and the consecutive 10 min-long actinic irradiation routine with 77 E m^−2^ s^−1^ (left charts). The test plants were cultured in either pure Steinberg medium (control), or Steinberg medium containing 10 g l^−1^ NaCl for 3 days, respectively. The photochemical [Y(II)], regulated non-photochemical [Y(NPQ)] and non-regulated non-photochemical [Y(NO)] quenching coefficients (right charts) were calculated using the basic, mutually independent chlorophyll fluorescence yields indicated in the top left subfigure. Figure S2: Measured and modelled responses of the assessed chlorophyll fluorescence induction parameters to 72 h-long Ni-treatments of the S. polyrhiza UD0401 clone, as compared to their respective control data. Cyrcles denote means (n = 4) of the repeated experiments (n = 3) at the applied concentrations. Thin black lines denote the best-fitting non-linear regression model for each ChlF parameter with 95% confidence intervals (gray shaded areas). Figure S3: Measured and modelled responses of the assessed chlorophyll fluorescence induction parameters to 72 h-long Cr(VI)-treatments of the S. polyrhiza UD0401 clone, as compared to their respective control data. Cyrcles denote means (n = 4) of the repeated experiments (n = 3) at the applied concentrations. Thin black lines denote the best-fitting non-linear regression model for each ChlF parameter with 95% confidence intervals (gray shaded areas). Figure S4: Measured and modelled responses of the assessed chlorophyll fluorescence induction parameters to 72 h-long NaCl-treatments of the S. polyrhiza UD0401 clone, as compared to their respective control data. Cyrcles denote means (n = 4) of the repeated experiments (n = 3) at the applied concentrations. Thin black lines denote the best-fitting non-linear regression model for each ChlF parameter with 95% confidence intervals (gray shaded areas).
