# Supplementary material for: Chlorophyll Fluorescence Imaging-Based Duckweed Phenotyping to Assess Acute Phytotoxic Effects
Source: Plants (Basel). 2021 Dec 14;10(12):2763. doi: 10.3390/plants10122763 (PMC8707530; doi:10.3390/plants10122763)

Figure S2. Kinetics in the chlorophyll fluorescence yield of the S. polyrhiza UD0401 clone during the first saturation pulse after 20 min of dark adaption, and the consecutive 10 min-long actinic irradiation routine with 77 μE m^{-2} s^{-1} (left charts). The test plants were cultured in either pure Steinberg medium (control), or Steinberg medium containing 10 g l^{-1} NaCl for 3 days, respectively. The photochemical [Y(II)], regulated non-photochemical [Y(NPQ)] and non-regulated non-photochemical [Y(NO)] quenching coefficients (right charts) were calculated using the basic, mutually independent chlorophyll fluorescence yields indicated in the top left subfigure.

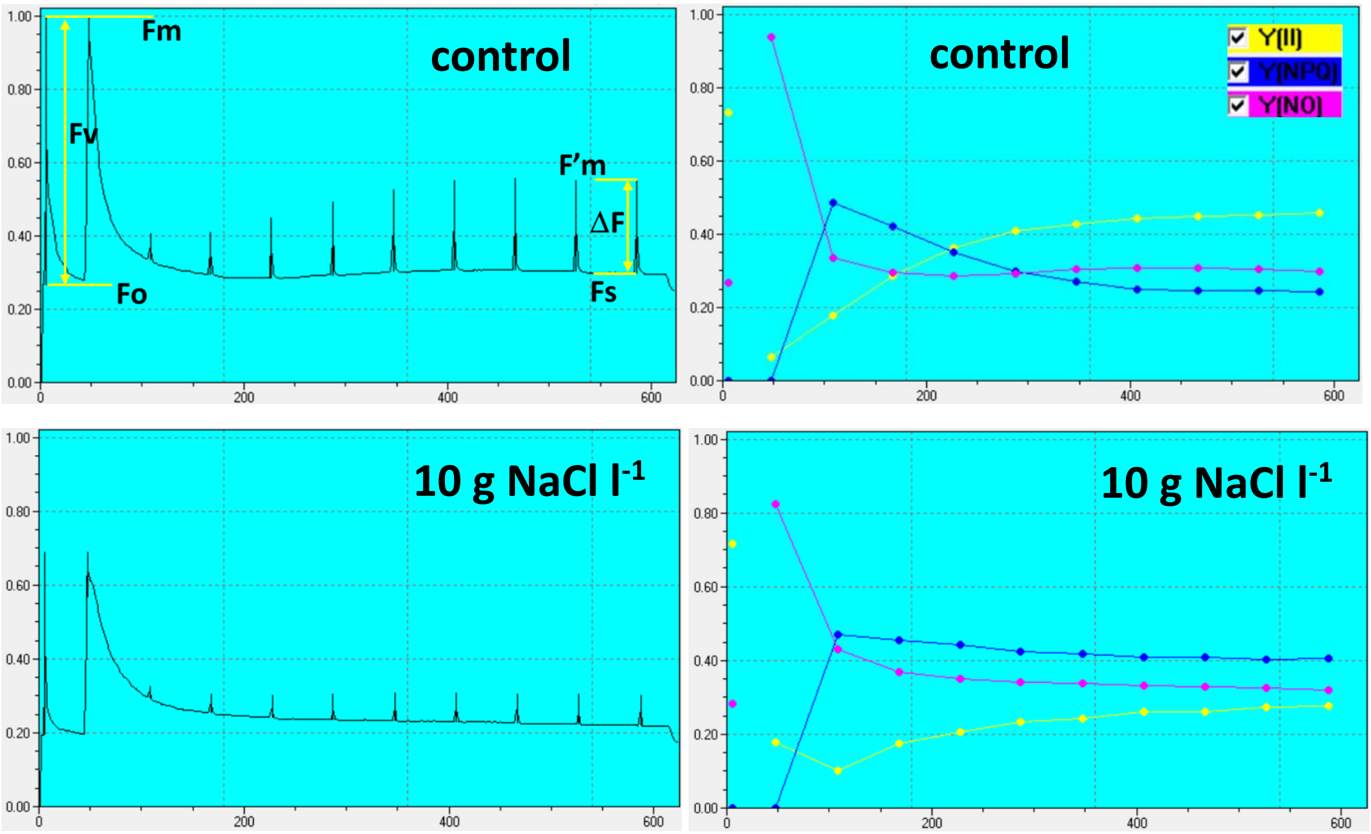

Supplement: Supplementary file 1 [file plants-10-02763-s001.zip › plants-1427447-supplementary/Figure S1.pdf]
