# Supplementary material for: Chlorophyll Fluorescence Imaging-Based Duckweed Phenotyping to Assess Acute Phytotoxic Effects
Source: Plants (Basel). 2021 Dec 14;10(12):2763. doi: 10.3390/plants10122763 (PMC8707530; doi:10.3390/plants10122763)

Figure S6. Measured and modelled responses of the assessed chlorophyll fluorescence induction parameters to 72 h-long Cr(VI)-treatments of the *S. polyrhiza* UD0401 clone, as compared to their respective control data. Cyrcles denote means (n=4) of the repeated experiments (n=3) at the applied concentrations. Thin black lines denote the best-fitting non-linear regression model for each ChlF parameter with 95% confidence intervals (gray shaded areas).

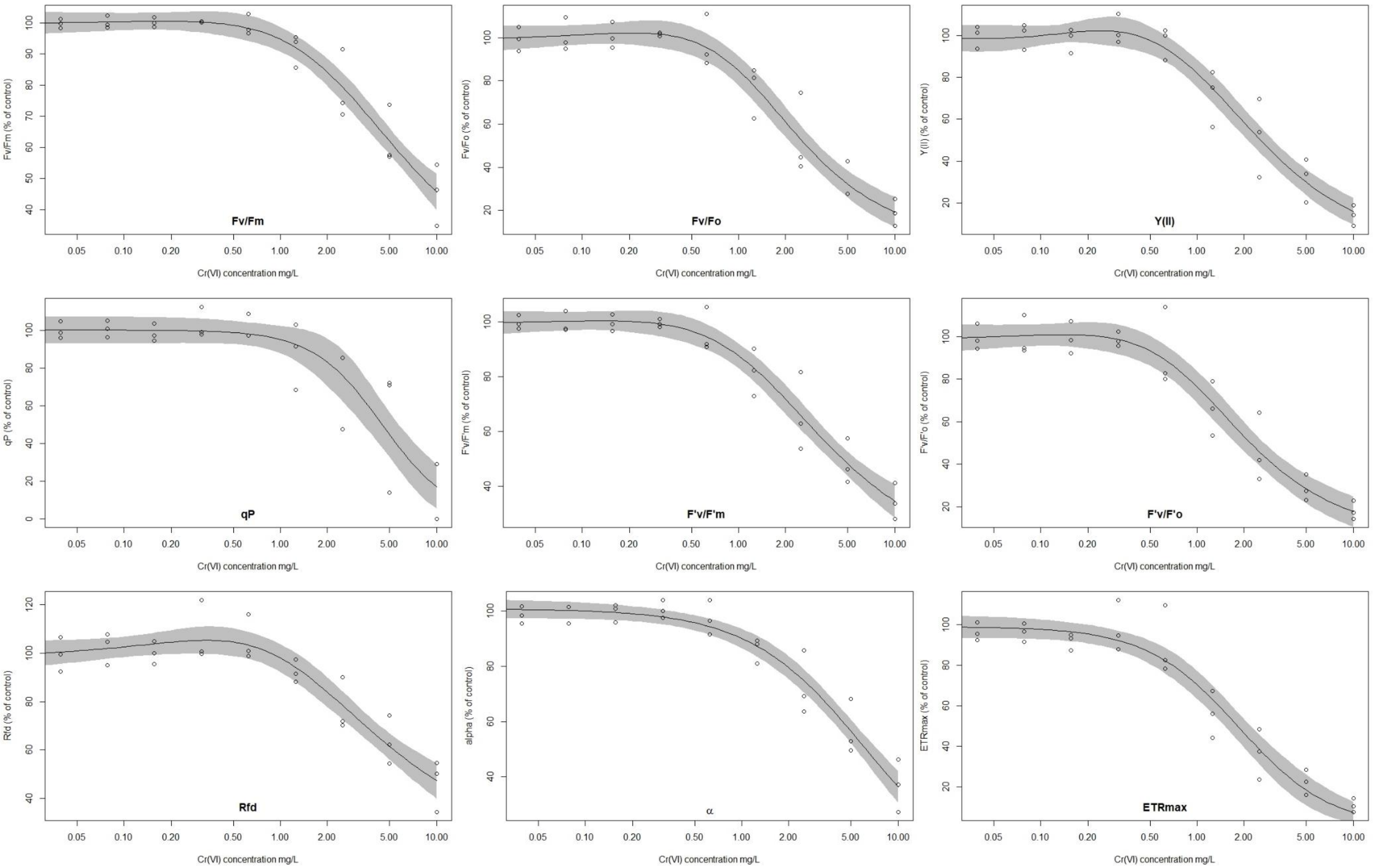

Supplement: Supplementary file 1 [file plants-10-02763-s001.zip › plants-1427447-supplementary/Figure S3.pdf]
