# Supplementary material for: Chlorophyll Fluorescence Imaging-Based Duckweed Phenotyping to Assess Acute Phytotoxic Effects
Source: Plants (Basel). 2021 Dec 14;10(12):2763. doi: 10.3390/plants10122763 (PMC8707530; doi:10.3390/plants10122763)
Supplement: Supplementary file 1 [file plants-10-02763-s001.zip › plants-1427447-supplementary/Table S1.pdf]

Supplementary

Table S1 Literature reports on the application of ChlF imaging method in duckweed ecotoxicology and ecophysiology studies.

**Species**	**Treatment**	**Exposure duration**	**ChlF-based endpoint (applied instrument, manufacturer)**	**Reference**
L. minor	linuron	90 min	Y(II), F'o (custom-built)	Hulsen et al. [65]
L. minor	paraquat, triclozan, alizarine	24 h	Y(II) (Imaging-PAM, Walz)	Küster et al. [66]
Lemna sp. (unspecified)	diuron, atrazine, simazine, glyphosate	7 d	Fo, Fm, Fv/Fm, NPQ, ETRmax (Imaging-PAM, Walz)	Kumar and Han [44]
L. gibba	CuO nanoparticles, Cu, polyacrylic acid	48 h	Fv/Fm, F'v/F'm, qP, qN, NPQ (Imaging-PAM, Walz)	Perreault et al. [45]
L. minor	Al nanoparticles	7 d	Fv/Fm, Y(II), qP, qN (Imaging-PAM, Walz)	Juhel et al. [67]
L. minor, L. gibba, Landoltia punctata	Zn	7 d	Fv/Fm (Imaging-PAM, Walz)	Lahive et al. [68]
L. minor, L. gibba, Landoltia punctata	Zn	7 d	Fv/Fm, Y(II), qP, NPQ (Imaging-PAM, Walz)	Lahive et al. [46]
L. paucicostata (recent name is *L. aequinoctialis*)	phenol	72 h	Fo, Fm, Fv/Fm, NPQ (Imaging-PAM, Walz)	Park et al. [52]
L. minor	fluoranthene	10 d	Fo, Fv/Fm, Y(II), NPQ, Rfd (FluorCam, PSI)	Zezulka et al. [69]
L. minor	electromagnetic radiation	24 h	Fv/Fm, Y(II), NPQ (FluorCam, PSI)	Senavirathna et al. [70]
L. minor	glyphosate	45 min	ETR (Imaging-PAM, Walz)	Gomes and Juneau [71]
L. minor	diclofenac, paracetamol	10 d	Fv/Fm, Y(II), NPQ, Rfd (FluorCam, PSI)	Kummerová et al. [53]
L. minor	Cd	72 h	Fv/Fm, Fv/Fo, Fo/Fv (FluorCam, PSI)	Pietrini et al. [43]
L. gibba	ibuprofen	8 d	Fo, Fm, Fv/Fm, Y(II), qP, NPQ (Imaging-PAM, Walz)	Di Baccio et al. [72]
L. minor, L. gibba, L. paucicostata (recent name is *L. aequinoctialis*)	atrazine, diuron, paraquat, simazine	72 h	Fv/Fm, Y(II), F'v/F'm, ETRmax (Imaging-PAM, Walz)	Park et al. [34]
L. gibba	Ag nanoparticles	7 d	Fv/Fm, qP, NPQ (Imaging-PAM, Walz)	Dewez et al. [48]
L. minor, L. minuta	light intensity	7 d	Fv/Fm, Y(II), qP, qN (Imaging-PAM, Walz)	Paolacci et al. [73]
L. minor, L. gibba	acetaminophen, chlorpromazine, diclofenac	4 d	Fv/Fm (FluorCam, PSI)	Alkimin et al. [56]
L. minor	ZnO nanoparticles	7 d	Fv/Fm (Imaging-PAM, Walz)	Chen et al. [74]

L. minor	Sulfamethoxazole, N4-acetylsulfamethoxazole	28 d	Fv/Fm, Y(II), NPQ, ETR (Imaging-PAM, Walz)	Grenni et al. [75]
L. minor	microplastics	7 d	Fv/Fm, Y(II), qP, qN (Imaging-PAM, Walz)	Mateos-Cárdenas et al. [76]
L. minor, L. gibba	ciprofloxacin	5 d	Fv/Fm (FluorCam, PSI)	Nunes et al. [77]
L. minor	perfluorooctanoic acid	7 d	Fv/Fm, Y(II), NPQ, ETR (Imaging-PAM, Walz)	Pietrini et al. [49]
L. aequinoctialis	heavy ion irradiation-induced mutagenesis	-	Fv/Fm, Y(II), qN (Imaging-PAM, Walz)	Liu et al. [78]
S. polyrhiza	paraquat	72 h	Fv/Fm, rETRmax, IK, α (Imaging-PAM, Walz)	Park et al. [50]
L. aequinoctialis, S. polyrhiza	Cu	7 d	Fv/Fm, Fv/Fo, Fo/Fv (Imaging-PAM, Walz)	Shi et al. [79]
L. minor	diuron	72 h	Fv/Fm, NPQ, ETRmax (Imaging-PAM, Walz)	Lee et al. [51]
L. minor	light intensity	5 d	Fv/Fm, Y(II), Y(NPQ), Y(NO), NPQ (Imaging-PAM, Walz)	Walsh et al. [80]
L. minor	plant density	7 d	Fv/Fm, Y(II), Y(NPQ), Y(NO) (Imaging-PAM, Walz)	Walsh et al. [81]
