# Supplementary material for: Chlorophyll Fluorescence Imaging-Based Duckweed Phenotyping to Assess Acute Phytotoxic Effects
Source: Plants (Basel). 2021 Dec 14;10(12):2763. doi: 10.3390/plants10122763 (PMC8707530; doi:10.3390/plants10122763)
Supplement: Supplementary file 1 [file plants-10-02763-s001.zip › plants-1427447-supplementary/Table S4.pdf]

Table S7. Summary statistics of the assessed chlorophyll fluorescence induction parameters after 72 h-long NaCl-treatments of the *S. polyrhiza* UD0401 clone. The table summarizes minimums (Min), maximums (Max), arithmetic means (Mean), standard deviations (SD) and coefficients of variation (CV) of pooled data, expressed as percentage of their respective control means, from 3 independent experiments with 4-4 parallel treatments at each applied NaCl concentraiton (n=12). Different upper indicate significantly ($p<0.05$) different medians for different concentrations according to the Kruskal-Wallis test and *post hoc* Mann-Whitney pairwise comparisons

**Concentration ($g\ l^{-1}$)**		**0**	**2**	**4**	**6**	**8**	**10**	**12**	**14**	**16**
**Sample size (n)**		12	12	12	12	12	12	12	12	12
**Fv/Fm**	**Min**	98.4	100.0	102.3	100.2	97.0	88.6	86.0	68.5	13.8
	**Max**	101.2	104.0	106.3	104.9	102.8	99.4	93.0	86.2	68.2
	**Mean**	100.0	101.5	104.6	103.1	100.3	94.9	89.0	75.3	41.0
	**SD**	0.8	1.1	1.0	1.4	2.0	3.3	2.7	5.9	19.1
	**CV**	0.8	1.1	1.0	1.4	2.0	3.4	3.0	7.8	46.7
	**Median**	100.0^{d}	101.4^{c}	104.9^{a}	103.7^{b}	100.3^{cd}	95.9^{e}	88.6^{f}	73.7^{g}	37.5^{h}
**Fv/Fo**	**Min**	94.3	100.0	109.1	100.7	89.9	67.4	62.6	36.7	4.3
	**Max**	104.6	116.7	127.1	121.1	111.3	97.7	77.9	62.5	36.4
	**Mean**	100.0	106.0	119.6	112.8	101.3	84.1	68.9	45.9	17.8
	**SD**	3.1	4.5	4.7	6.1	7.6	9.1	5.9	8.4	11.4
	**CV**	3.1	4.3	3.9	5.4	7.5	10.8	8.5	18.2	63.8
	**Median**	99.9^{d}	105.1^{c}	120.5^{a}	114.7^{b}	101.0^{cd}	86.1^{e}	68.3^{f}	43.4^{g}	13.9^{h}
**Y(II)**	**Min**	94.4	98.3	104.6	83.9	81.6	54.5	35.2	26.5	0.0
	**Max**	104.5	112.2	115.5	112.0	100.1	77.5	62.4	52.9	40.5
	**Mean**	100.0	104.7	111.0	101.6	87.8	66.7	50.1	42.1	14.6
	**SD**	3.0	4.2	3.6	7.8	6.1	7.3	9.0	7.9	18.5
	**CV**	3.0	4.0	3.3	7.7	7.0	10.9	18.0	18.7	126.2
	**Median**	99.8^{c}	104.7^{b}	112.4^{a}	102.3^{bc}	85.9^{d}	67.8^{e}	50.9^{f}	42.0^{g}	0.0^{h}
**qP**	**Min**	96.7	99.2	96.6	80.5	81.6	60.8	46.2	0.0	0.0
	**Max**	102.0	106.6	103.2	101.5	96.3	82.7	71.1	69.5	65.8
	**Mean**	100.0	102.6	100.1	93.9	88.1	75.0	60.5	55.7	21.1
	**SD**	1.5	1.9	2.1	5.5	5.4	6.9	7.8	18.4	31.1
	**CV**	1.5	1.8	2.1	5.9	6.2	9.2	12.9	33.0	147.8
	**Median**	100.3^{b}	102.5^{a}	99.5^{b}	94.1^{c}	86.3^{d}	77.9^{e}	61.8^{f}	59.9^{f}	0.0^{g}
**Fv'/Fm'**	**Min**	96.6	96.9	107.0	102.4	95.9	82.2	70.7	58.3	12.9
	**Max**	103.0	107.7	113.8	114.1	104.2	94.3	88.4	82.8	64.4
	**Mean**	100.0	102.0	110.7	108.0	99.7	88.6	82.2	70.4	38.9
	**SD**	2.2	3.0	2.2	3.7	2.4	3.3	5.6	7.5	18.3
	**CV**	2.2	3.0	2.0	3.4	2.4	3.7	6.8	10.6	47.1
	**Median**	100.6^{bc}	102.1^{b}	110.8^{a}	108.2^{a}	99.6^{c}	88.1^{d}	82.0^{e}	68.4^{f}	35.8^{g}
**Fv'/Fo'**	**Min**	91.9	93.0	119.3	106.1	90.8	65.6	49.3	36.0	5.9
	**Max**	107.4	120.5	140.5	142.0	110.2	87.5	76.4	66.0	42.2
	**Mean**	100.0	105.2	130.4	121.9	99.2	76.4	66.0	50.3	22.5
	**SD**	5.2	7.7	6.6	11.3	5.6	6.0	8.7	9.4	13.1
	**CV**	5.2	7.3	5.1	9.2	5.6	7.9	13.2	18.7	58.5
	**Median**	101.3^{bc}	104.9^{b}	129.8^{a}	122.0^{a}	98.7^{c}	74.9^{d}	65.4^{e}	46.9^{f}	18.8^{g}
**Rfd**	**Min**	96.2	97.9	94.5	82.0	77.3	67.5	49.4	39.6	12.1
	**Max**	102.1	111.6	110.6	99.3	98.8	89.7	75.7	62.3	42.9
	**Mean**	100.0	106.9	100.8	91.6	90.1	81.4	64.9	47.3	24.6
	**SD**	1.7	3.3	4.5	6.0	7.6	7.5	8.3	6.8	10.0
	**CV**	1.7	3.1	4.5	6.5	8.4	9.3	12.7	14.4	40.6
	**Median**	100.6^{b}	107.8^{a}	99.6^{b}	90.4^{c}	92.9^{c}	83.8^{d}	62.3^{e}	45.3^{f}	22.2^{g}
**alpha**	**Min**	98.0	98.3	104.1	101.3	96.0	86.1	73.4	57.0	0.0
	**Max**	101.6	106.1	108.8	108.8	105.2	98.3	84.7	78.0	60.0
	**Mean**	100.0	101.5	106.7	104.8	100.0	91.0	79.4	64.8	32.3
	**SD**	1.3	2.3	1.3	2.3	2.6	3.7	3.0	7.2	21.0
	**CV**	1.3	2.3	1.2	2.2	2.6	4.1	3.8	11.1	65.0
	**Median**	99.7^{c}	101.3^{c}	106.8^{a}	105.1^{b}	99.9^{c}	90.8^{d}	79.0^{e}	63.2^{f}	30.8^{g}

ETR_{max}	**Min**	85.1	87.8	101.2	60.6	58.9	35.0	23.1	17.5	0.0
	**Max**	117.1	127.4	129.1	138.3	84.2	56.6	38.8	33.7	23.9
	**Mean**	100.0	105.1	119.4	98.7	67.4	43.3	31.9	25.0	11.0
	**SD**	10.1	13.1	7.4	20.2	6.4	5.6	5.6	4.9	9.0
	**CV**	10.1	12.5	6.2	20.4	9.4	13.0	17.5	19.5	81.7
	**Median**	98.9[b]	103.3[b]	120.8[a]	98.5[b]	67.2[c]	42.6[d]	31.8[e]	23.9[f]	10.1[g]
