# Supplementary material for: Chlorophyll Fluorescence Imaging-Based Duckweed Phenotyping to Assess Acute Phytotoxic Effects
Source: Plants (Basel). 2021 Dec 14;10(12):2763. doi: 10.3390/plants10122763 (PMC8707530; doi:10.3390/plants10122763)
Supplement: Supplementary file 1 [file plants-10-02763-s001.zip › plants-1427447-supplementary/Table S5.pdf]

Tables S9. Summary statistics of the assessed basic chlorophyll fluorescence induction parameters after 72 h-long treatments of the *S. polyrhiza* UD0401 clone to Ni, Cr(VI) and NaCl, respectively. The table summarizes minimums (Min), maximums (Max), arithmetic means (Mean), standard deviations (SD) and coefficients of variation (CV) of pooled data expressed as percentage of their respective control means, from 3 independent experiments with 4-4 parallel treatments at each applied concentration (n=12). Different upper cases indicate significantly ($p<0.05$) different medians for different concentrations according to the Kruskal-Wallis test and *post hoc* Mann-Whitney pairwise comparisons.

Ni-treatments

**Concentration (mg l^{-1})**		**0**	**0.039**	**0.078**	**0.156**	**0.313**	**0.625**	**1.25**	**2.5**	**5**	**10**
**Sample size (n)**		12	12	12	12	12	12	12	12	12	12
**Fo**	**Min**	81.0	72.4	80.6	72.6	78.1	73.2	80.4	95.4	102.8	103.5
	**Max**	119.0	134.8	118.1	114.5	116.9	118.0	143.3	131.9	159.9	210.7
	**Mean**	100.0	94.4	96.0	93.5	91.6	86.3	107.4	111.9	126.7	150.6
	**SD**	13.2	17.4	13.5	9.9	11.7	11.9	19.8	11.1	18.8	32.4
	**CV**	13.2	18.4	14.1	10.5	12.7	13.8	18.4	9.9	14.9	21.5
	**Median**	98.0^{c}	93.5^{cd}	90.2^{cd}	93.7^{c}	86.8^{cd}	83.2^{d}	102.1^{c}	112.3^{c}	121.1^{b}	140.0^{a}
**Fm**	**Min**	81.6	72.8	83.1	73.8	78.7	74.3	77.7	69.8	57.5	59.0
	**Max**	121.2	126.6	116.6	120.4	105.6	117.1	114.0	88.3	92.8	111.0
	**Mean**	100.0	93.1	95.7	95.9	91.0	86.0	92.0	78.7	74.9	79.5
	**SD**	13.5	14.9	11.2	11.8	9.5	11.8	12.2	5.4	11.3	15.9
	**CV**	13.5	16.0	11.7	12.3	10.5	13.7	13.2	6.8	15.2	20.0
	**Median**	97.1^{a}	93.4^{ab}	92.8^{a}	96.9^{a}	86.6^{ab}	82.0^{b}	89.7^{ab}	79.0^{b}	75.2^{c}	75.9^{bc}
**Fv**	**Min**	81.8	73.0	82.3	74.2	77.8	71.3	67.6	54.9	37.1	38.5
	**Max**	122.6	123.6	117.8	122.5	106.6	116.8	112.0	80.3	67.6	72.6
	**Mean**	100.0	92.6	95.6	96.7	90.9	85.9	86.2	66.2	55.4	52.7
	**SD**	13.7	14.1	10.8	12.6	9.2	11.9	12.5	6.9	10.2	10.8
	**CV**	13.7	15.2	11.3	13.0	10.2	13.9	14.5	10.4	18.4	20.6
	**Median**	96.9^{a}	92.8^{ab}	93.2^{a}	98.4^{a}	86.3^{ab}	82.2^{b}	83.7^{b}	64.0^{c}	56.8^{d}	50.8^{d}
**Fs**	**Min**	82.1	73.7	79.7	75.5	77.7	73.2	80.1	79.5	77.3	84.3
	**Max**	120.1	121.5	110.7	115.4	106.6	116.8	125.3	109.0	118.7	149.8
	**Mean**	100.0	91.2	92.3	95.1	91.0	84.4	102.1	94.6	96.1	111.5
	**SD**	12.7	13.2	10.5	10.7	11.0	11.9	14.7	8.1	14.1	20.5
	**CV**	12.7	14.5	11.3	11.2	12.1	14.1	14.4	8.6	14.7	18.4
	**Median**	97.6^{ab}	92.7^{bc}	88.1^{b}	96.4^{ab}	86.0^{bc}	80.5^{c}	99.0^{ab}	95.2^{b}	95.5^{ab}	102.8^{a}
**Fm'**	**Min**	83.5	73.8	83.2	75.6	80.8	75.2	66.1	56.1	47.8	52.0
	**Max**	124.3	118.9	112.4	122.4	110.1	122.9	110.1	80.6	84.6	97.8
	**Mean**	100.0	92.2	94.7	97.9	94.4	88.3	87.4	68.2	64.8	72.3
	**SD**	13.8	12.2	9.6	12.8	10.0	13.7	14.5	8.0	11.7	14.7
	**CV**	13.8	13.3	10.2	13.0	10.6	15.5	16.6	11.7	18.0	20.3
	**Median**	98.1^{a}	93.0^{ab}	93.5^{ab}	99.0^{ab}	91.1^{ab}	82.6^{b}	89.5^{ab}	67.8^{c}	63.8^{c}	65.7^{c}
**ΔF'**	**Min**	83.3	74.0	86.0	75.6	84.7	73.5	47.7	19.7	10.7	11.8
	**Max**	129.4	114.7	116.1	130.7	120.1	130.9	96.4	46.8	31.1	29.0
	**Mean**	100.0	93.5	97.8	101.8	99.6	94.2	66.5	32.0	21.5	18.1
	**SD**	15.5	11.3	10.2	16.0	10.7	17.2	17.0	9.1	7.2	5.6
	**CV**	15.5	12.1	10.4	15.8	10.7	18.3	25.6	28.5	33.4	30.8
	**Median**	97.8^{a}	95.9^{a}	95.9^{a}	101.8^{a}	99.8^{a}	88.4^{a}	63.6^{b}	29.3^{c}	23.2^{d}	16.8^{e}

Cr(VI)-treatments

Concentration (mg l^{-1})		**0**	**0.039**	**0.078**	**0.156**	**0.313**	**0.625**	**1.25**	**2.5**	**5**	**10**
**Sample size (n)**		12	12	12	12	12	12	12	12	12	12
**Fo**	**Min**	84.3	73.3	66.3	74.5	69.1	61.9	90.4	109.3	119.9	144.8
	**Max**	110.8	113.1	112.8	127.8	134.1	142.5	171.2	176.3	216.5	234.1
	**Mean**	100.0	92.9	91.5	98.2	100.6	105.7	125.7	141.0	155.6	184.0
	**SD**	7.8	10.4	13.7	14.8	17.2	19.4	23.9	23.6	27.3	31.5
	**CV**	7.8	11.2	15.0	15.1	17.1	18.3	19.0	16.7	17.6	17.1
	**Median**	100.2^{f}	94.2^{g}	92.3^{fg}	92.6^{efg}	98.9^{ef}	106.5^{e}	124.0^{cd}	139.5^{bc}	150.1^{b}	188.2^{a}
**Fm**	**Min**	85.1	74.6	73.5	73.3	71.6	68.9	83.4	64.8	62.2	62.3
	**Max**	112.8	107.2	107.7	127.6	134.0	124.9	126.4	125.3	95.7	88.8
	**Mean**	100.0	92.4	91.5	98.7	101.5	102.4	102.8	92.5	79.0	75.9
	**SD**	7.7	9.6	10.9	14.8	15.8	13.8	13.7	17.4	11.0	8.5
	**CV**	7.7	10.4	11.9	15.0	15.6	13.5	13.3	18.8	14.0	11.2
	**Median**	99.7^{ab}	93.3^{b}	90.6^{b}	96.1^{ab}	100.2^{ab}	102.5^{a}	102.0^{a}	96.7^{ab}	80.7^{c}	73.5^{c}
**Fv**	**Min**	85.4	75.1	74.6	72.9	72.5	71.6	80.7	43.1	33.5	24.2
	**Max**	113.6	105.2	106.5	127.5	134.0	118.5	118.4	115.1	68.6	49.0
	**Mean**	100.0	92.2	91.5	98.8	101.8	101.2	94.0	73.9	49.8	34.7
	**SD**	7.8	9.6	10.3	15.0	15.5	12.6	12.4	20.9	11.5	7.6
	**CV**	7.8	10.4	11.3	15.2	15.2	12.5	13.2	28.3	23.1	21.9
	**Median**	100.5^{ab}	92.3^{bc}	90.9^{c}	97.8^{abc}	101.6^{ab}	99.7^{a}	91.1^{abc}	75.0^{d}	50.1^{e}	33.3^{f}
**Fs**	**Min**	87.5	71.0	69.1	75.2	71.4	68.3	88.2	80.0	86.5	82.3
	**Max**	112.5	111.7	101.6	126.0	132.5	127.4	130.5	132.5	145.6	163.9
	**Mean**	100.0	93.1	90.0	98.6	97.0	99.1	108.6	109.8	106.9	123.9
	**SD**	7.2	12.1	10.0	14.3	17.6	15.1	13.5	18.3	18.1	24.8
	**CV**	7.2	13.0	11.2	14.5	18.1	15.3	12.4	16.7	16.9	20.0
	**Median**	99.1^{b}	95.4^{bc}	92.7^{c}	96.4^{bc}	92.9^{bc}	98.4^{bc}	107.2^{ab}	109.7^{ab}	105.4^{ab}	126.7^{a}
**Fm’**	**Min**	86.0	72.4	74.5	71.0	68.3	72.4	69.7	54.5	52.5	49.5
	**Max**	115.9	108.4	103.0	127.8	133.5	115.0	113.3	107.6	95.0	97.2
	**Mean**	100.0	92.7	90.0	97.3	98.8	96.3	89.5	81.1	69.9	74.2
	**SD**	7.6	10.6	8.9	15.6	16.8	12.7	15.3	18.4	13.1	14.5
	**CV**	7.6	11.4	9.9	16.0	17.0	13.2	17.1	22.6	18.8	19.5
	**Median**	100.1^{a}	93.0^{ab}	89.0^{b}	94.9^{ab}	97.3^{ab}	92.8^{ab}	87.3^{ab}	86.2^{bc}	71.8^{c}	76.0^{c}
**ΔF’**	**Min**	84.1	72.9	75.3	65.6	64.6	74.1	33.8	12.1	8.9	7.3
	**Max**	120.4	109.4	110.2	130.1	134.6	115.3	92.2	77.1	35.5	18.0
	**Mean**	100.0	92.2	90.0	95.6	101.2	93.0	65.0	44.3	22.7	10.5
	**SD**	8.8	10.8	10.7	18.5	18.0	12.9	20.3	21.2	9.6	3.8
	**CV**	8.8	11.8	11.9	19.4	17.8	13.8	31.2	47.9	42.2	36.4
	**Median**	100.4^{a}	93.7^{ab}	88.9^{b}	91.8^{ab}	99.9^{ab}	93.3^{ab}	63.8^{c}	48.5^{d}	25.2^{e}	8.8^{f}

NaCl-treatments

Concentration (g l^{-1})		**0**	**2**	**4**	**6**	**8**	**10**	**12**	**14**	**16**
**Sample size (n)**		12	12	12	12	12	12	12	12	12
**Fo**	**Min**	84.3	69.6	60.3	63.4	59.6	69.6	60.3	50.5	46.7
	**Max**	117.5	103.4	99.1	97.7	104.4	105.5	92.3	88.2	86.8
	**Mean**	100.0	87.3	74.1	83.7	81.5	80.9	74.7	72.5	68.3
	**SD**	8.8	11.3	13.6	11.3	13.8	11.8	11.5	10.0	13.6
	**CV**	8.8	13.0	18.4	13.5	17.0	14.6	15.3	13.8	19.9
	**Median**	99.1^{a}	88.1^{b}	72.0^{cd}	84.4^{bcd}	80.8^{bcd}	77.8^{bcd}	76.4^{cd}	71.1^{d}	68.7^{d}
**Fm**	**Min**	85.1	76.7	69.7	70.8	58.5	52.9	46.1	33.0	14.4
	**Max**	121.2	107.0	108.5	109.5	97.9	96.9	71.6	60.6	42.3
	**Mean**	100.0	91.0	84.2	91.6	82.1	71.8	57.7	44.1	27.6
	**SD**	9.8	10.7	13.4	13.4	14.0	13.6	8.7	9.1	8.5
	**CV**	9.8	11.8	15.9	14.6	17.0	18.9	15.1	20.7	30.6
	**Median**	98.7^{a}	89.8^{ab}	84.1^{b}	94.0^{ab}	84.1^{bc}	68.7^{cd}	57.8^{d}	41.2^{e}	27.8^{f}
**Fv**	**Min**	85.4	77.3	73.3	73.6	58.0	46.8	40.7	24.9	2.0
	**Max**	122.6	108.3	112.7	113.8	99.0	93.8	66.5	52.2	27.2
	**Mean**	100.0	92.3	88.0	94.5	82.3	68.4	51.4	33.6	12.5
	**SD**	10.3	10.6	13.3	14.3	14.4	14.6	8.3	9.5	8.7
	**CV**	10.3	11.5	15.1	15.2	17.5	21.3	16.2	28.2	69.9
	**Median**	98.9^{a}	90.4^{abc}	88.2^{bc}	97.7^{ab}	86.0^{c}	64.7^{d}	51.3^{e}	29.6^{f}	8.5^{g}
**Fs**	**Min**	87.5	71.0	68.0	76.5	66.2	67.2	62.4	55.0	37.9
	**Max**	120.1	102.9	109.9	113.0	105.2	106.1	95.5	96.1	81.1
	**Mean**	100.0	86.8	84.1	97.5	88.3	82.5	77.0	70.2	58.7
	**SD**	9.2	10.5	15.2	13.3	12.8	12.6	11.1	11.9	13.1
	**CV**	9.2	12.1	18.1	13.6	14.5	15.2	14.4	16.9	22.4
	**Median**	98.1^{a}	88.2^{bc}	84.3^{bcd}	101.4^{a}	90.5^{abc}	80.6^{cd}	73.0^{de}	69.1^{ef}	62.1^{f}
**Fm’**	**Min**	86.0	70.7	76.5	67.6	59.3	51.3	45.6	35.7	20.2
	**Max**	124.3	102.3	118.2	120.2	97.8	86.4	67.9	69.6	54.5
	**Mean**	100.0	90.0	91.8	98.9	80.1	65.0	54.9	48.2	35.7
	**SD**	11.2	10.0	14.4	15.4	11.2	10.4	7.3	9.9	9.7
	**CV**	11.2	11.1	15.7	15.6	14.0	16.0	13.2	20.4	27.2
	**Median**	99.4^{a}	90.4^{a}	92.5^{ab}	102.7^{a}	80.1^{b}	62.1^{c}	53.2^{d}	46.5^{e}	35.2^{f}
**ΔF’**	**Min**	83.3	70.2	86.4	56.7	51.1	31.4	16.5	9.5	-3.3
	**Max**	129.4	107.8	128.4	128.7	91.3	65.5	35.6	34.6	22.0
	**Mean**	100.0	93.9	101.3	100.8	70.2	43.4	27.5	20.8	7.0
	**SD**	14.0	10.7	13.7	19.7	10.9	9.7	6.3	7.6	8.3
	**CV**	14.0	11.4	13.5	19.6	15.5	22.4	22.9	36.7	119.6
	**Median**	100.4^{a}	94.8^{a}	101.0^{a}	99.8^{a}	68.6^{b}	40.2^{c}	29.3^{d}	19.3^{e}	4.7^{f}
